# Green Extraction of Polyphenols from *Elaeagnus angustifolia* L. Using Natural Deep Eutectic Solvents and Evaluation of Bioactivity

**DOI:** 10.3390/molecules29112412

**Published:** 2024-05-21

**Authors:** Lu Li, Jingjing Lv, Xiaoqin Wang, Xiujun Li, Dongqi Guo, Liling Wang, Na Zhang, Qinghua Jia

**Affiliations:** 1 College of Food Science and Engineering, Tarim University, Alar 843300, China; lilu9292jn@163.com (L.L.);; 2 Production & Construction Group Key Laboratory of Special Agricultural Products Further Processing in Southern Xinjiang, Alar 843300, China; 3 Analysis and Testing Center, Tarim University, Alar 843300, China

**Keywords:** *Elaeagnus angustifolia* L., natural deep eutectic solvent, polyphenols, green extraction, UPLC–IMS–QTOF–MS

## Abstract

In the study, natural deep eutectic solvents (NADESs) were used as alternatives to traditional chemical solvents for the extraction of polyphenols from *Elaeagnus angustifolia* L. Nine NADESs were tested for the first time and compared with ethanol and water (traditional solvents) regarding the extraction of phenolic compounds from *E. angustifolia* L. These solvents were particularly effective at extracting polyphenols, whose low water solubility usually requires high amounts of organic solvents. The solvent based on choline chloride and malonic acid provided optimal results and was selected for further optimization. The effects of material-to-liquid ratio, ultrasound time, and ultrasound temperature on the extraction efficiency were studied through single-factor experiments. These parameters were optimized by Box–Behnken design using response surface methodology. The optimal conditions identified were 49.86 g/mL of material-to-liquid ratio, 31.10 min of ultrasound time, and 62.35 °C of ultrasound temperature, resulting in a high yield of 140.30 ± 0.19 mg/g. The results indicated that the NADES extraction technique provided a higher yield than the conventional extraction process. The antioxidant activity of the extract of polyphenols from *E. angustifolia* L. was determined, and UPLC–IMS–QTOF–MS was used to analyze the phenolic compounds in it. The results revealed that the scavenging ability of 1,1-diphenyl-2-picryl-hydrazil and 2,2′-azinobis-(3-ethylbenzthiazoline-6-sulphonate) extracted by NADES was higher than that of polyphenols extracted by water and ethanol. Furthermore, a total of 24 phenolic compounds were identified in the extract. To the best of our knowledge, this is the first study in which a green and efficient NADES extraction method has been used to extract bioactive polyphenols from *E. angustifolia* L., which could provide potential value in pharmaceuticals, cosmetics, and food additives.

## 1. Introduction

*Elaeagnus angustifolia* L. is a deciduous tree belonging to the *Elaeagnacea* family. For wind breaks and sand fixation, *E. angustifolia* L. is mainly planted in the western regions of China [1]. However, *E. angustifolia* L. is also a relevant traditional medicine used for its medicinal benefits by Xinjiang local doctors. For example, the flowers of *E. angustifolia* L. were used for thoracalgia and asthma in Chinese *Uygur* medicine [2]. In addition, *Elaeagnus* plants contain a myriad of bioactive compounds [3,4], such as flavonoids, phenolic carboxylic acids, polyphenols, terpenoids, alkaloids, and steroids, responsible for the significant biological characteristics of plants, including antioxidant, antibacterial, analgesic, and anti-inflammatory effects [5,6].

In recent years, many researchers have focused on optimizing the extraction process and functional activity of polysaccharides [7,8,9,10] and seed oil [11,12] from *E. angustifolia* L. However, there is relatively little research on optimizing the extraction of polyphenols from *E. angustifolia* L. Faramarz et al. [13] and Saboonchian et al. [14] studied the total phenolic and flavonoid content of *E. angustifolia* L., and the results demonstrated that *E. angustifolia* L. contains high levels of phenolic and flavonoid compounds. Therefore, the optimization and utilization of polyphenols in *E. angustifolia* L. are particularly relevant. Cha Pei et al. [15] used ultrasonic extraction to extract polyphenols from *E. angustifolia* L. through single-factor experiments and orthogonal experiments. The optimal extraction process conditions were determined to be using 50% ethanol solution as the extraction solvent, with a material-to-liquid ratio of 1:12 and an acetone concentration of 50%, each time for 30 min, for a total of three times of extraction. Traditional extraction solvents, such as methanol, ethanol, and acetone, are often used as polyphenol extraction solvents. However, traditional extraction solvents have high volatility, are non-biodegradable, toxic, and pollute the environment [16]. First, we should currently consider how to extract polyphenols from *E. angustifolia* L. quickly, efficiently, and greenly. Natural deep eutectic solvents (NADESs) are generally environmentally friendly, low-cost solvents with low toxicities, in addition to being recyclable and biodegradable [17]. NADESs are composed of biodegradable components such as amino acids, organic acids, and sugars, which are more environmentally friendly and biocompatible than traditional solvents. This reduces the risk of adverse effects on health and environmental pollution [18,19]. Second, NADESs have lower volatility and higher boiling points, which means that they are less likely to evaporate during the extraction process [20]. This feature improves safety and enhances the efficiency of the extraction process by reducing exposure to volatile organic compounds. In addition, NADES could be easily prepared and recovered, making it a cost-effective and sustainable alternative to traditional solvents [21]. This was all on account of their advantages.

This study reported for the first time the green extraction of polyphenols from *E. angustifolia* L. using NADES and explored the optimal extraction conditions. To further optimize the extraction efficiency, response surface methodology (RSM) was used to optimize the extraction process, saving solvents and improving the extraction rate of polyphenols from *E. angustifolia* L. We also evaluated the antioxidant properties of NADES compared with traditional solvents, such as water and ethanol, for extracting polyphenols from *E. angustifolia* L. In addition, we used UPLC–IMS–QTOF–MS to identify phenolic compounds from *E. angustifolia* L.

## 2. Results and Discussion

### 2.1. Selection of Extraction Solvent

Solvent is a particularly relevant factor after the fixed extraction method. The properties of solvents and solutes are closely related to their interactions, and their influence on extraction yield should not be underestimated [22]. In general, NADESs fall into different primary forms, which are composed of different HBAs and HBDs. Organic acids, polyols, and amides are most commonly used as HBDs, whereas choline chloride, l-proline, and betaine are often used as HBAs [23]. Due to their inexpensive initial components, simple manufacturing, interaction with water, low viscosity, and excellent biodegradability, choline chloride and different HBDs, including carboxylic acids, alcohols, and amides, are the most popular in processing [24,25].

**Figure 1:**
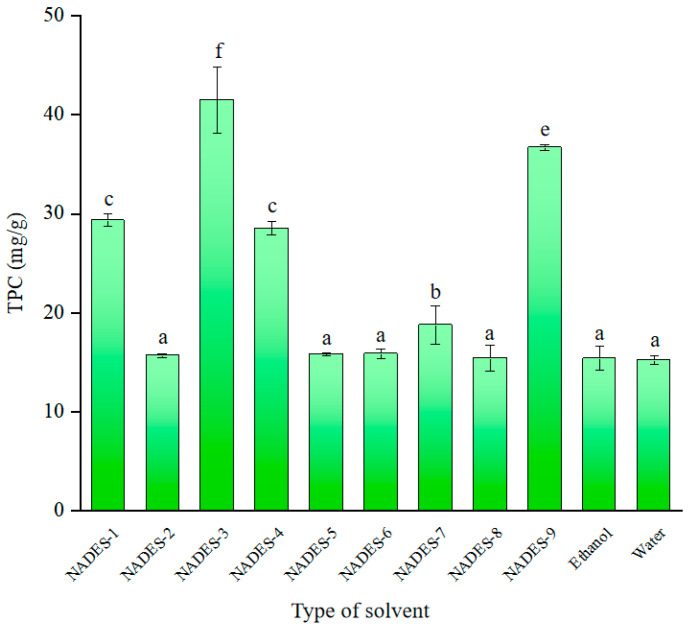
Effects of different NADES, ethanol, and water on the extraction yield. (Different letters represent significant differences, *p* < 0.05).

In the current study, we screened in related studies NADESs [26] for efficiently extracting nine polyphenols from *E. angustifolia* L. and the results are shown in Figure 1. NADES1–NADES9 exhibited significantly higher extraction rates than ethanol and water, with NADES3 showing the best extraction efficiency (41.49 ± 1.28 vs. 15.39 ± 1.21 vs. 15.25 ± 0.47 mg/g), which was consistent with the results of the previous study by Wei Wang et al. [27]. In the present study, the soluble polyphenols in *E. angustifolia* L. were polar substances. According to the principle of similarity and compatibility, using HBAs and HBDs to make NADESs was more suitable for extracting polar substances. Ethanol or water solvents have lower electrostatic and van der Waals interactions; thus, the interaction between ethanol or water and *E. angustifolia* L. was unfavorable [28]. In addition, as shown in Figure 1, the extraction efficiency varies among different solvents, with NADES3 having the highest extraction efficiency, followed by NADES7 and NADES1 (36.67 ± 0.31 and 29.34 ± 0.62 mg/g). Zhen [29] investigated the impact of varying HBD and HBA combinations on the interaction between the target compound and NADES during the extraction process, which in turn affects their solubility and ability to dissolve polyphenols. This study suggests that the intermolecular forces generated by NADES3 are particularly conducive to the extraction of phenolic compounds from *E. angustifolia* L.

### 2.2. Single-Factor Experiment

**Figure 2:**
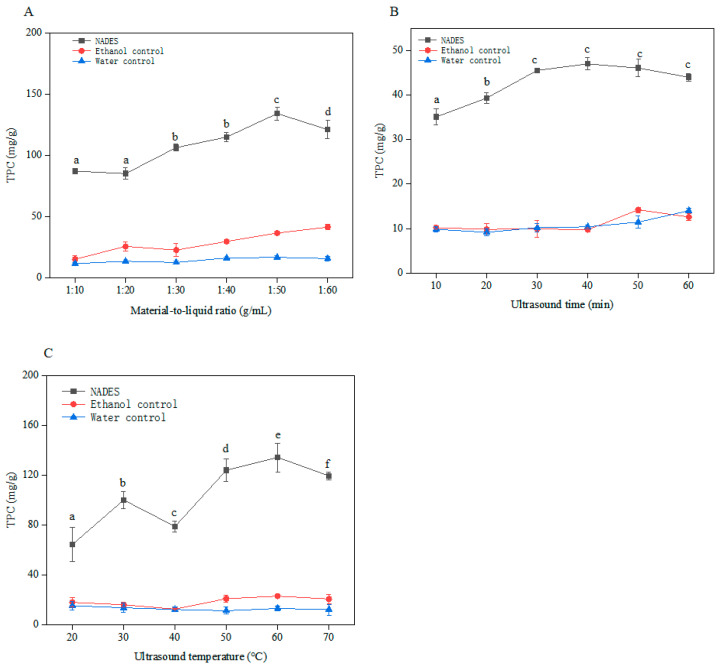
Impact of various operating parameters on total phenolic content (TPC): material-to-liquid ratio (**A**), ultrasound time (**B**), and ultrasound temperature (**C**). (Different letters represent significant differences, *p* < 0.05).

The molecules of HBD and HBA are broken down by high-frequency sound waves, and the application of ultrasound could make certain components more soluble, resulting in the production of more stable and homogeneous mixtures [30]. To further improve the extraction rate, we analyzed the effects of ultrasound time, ultrasound temperature, and material-to-liquid ratio on the extraction process of NADES. As shown in Figure 2A–C, under the same conditions, the TPC value extracted by NADES was significantly higher than that extracted from *E. angustifolia* L. by water and ethanol among the three single factors, suggesting that the extraction efficiency of NADES was high. Below, we would analyze each single factor involved in NADES extraction of *E. angustifolia* L. polyphenols in sequence.

#### 2.2.1. Effect of Material-to-Liquid Ratio

This experiment was performed to study the effects of the material-to-liquid ratio on the TPC value of *E. angustifolia* L. (Figure 2A). The TPC value gradually increased as the material-to-liquid ratio increased. The maximum value of 134.08 mg/g was reached at a material-to-liquid ratio of 1:50 g/mL. The material-to-liquid ratio has an impact on the extraction efficiency, similar to the study by Jiao et al. [31]. Subsequently, the TPC value showed a downward trend. The possible reason for this may be that under low material-to-liquid ratios, elevating this material-to-liquid ratio heightens the interface between the solute and solvent and accelerates the velocity of ultrasonic propagation, consequently enhancing the dissolution rate [32]. Additionally, the cavitation phenomenon is due to the intensified ultrasonic cavitation that arises at a higher material-to-liquid ratio [33,34]. The formation of tiny bubbles hinders the contact between polyphenols and solvents and the propagation of ultrasound, thereby reducing the dissolution efficiency. Thus, 1:50 g/mL was selected as the most suitable material-to-liquid ratio.

#### 2.2.2. Effect of Ultrasound Time

The impact of the ultrasound time on the TPC value was analyzed (Figure 2B). The TPC value increased over time, hitting its peak at 30 min at 45.53 mg/g. Following this, the TPC value gradually fell. Because most of the polyphenols had already been extracted within 30 min. It was reported that prolonged ultrasound time can also lead to the degradation or oxidation of polyphenols, resulting in a decrease in extraction efficiency [35]. This result was in agreement with those presented in previous research reports [36]. Thus, 30 min was selected as the most suitable extraction time for our experiment.

#### 2.2.3. Effect of Ultrasound Temperature

This experiment was conducted to assess the influences of ultrasound temperature on the TPC value of *E. angustifolia* L. (Figure 2C). The TPC value gradually increased as the temperature rose from 20 °C to 60 °C. The maximum TPC value has reached 134.16 mg/g. However, when the temperature is 70 °C, the TPC value decreases, which is consistent with the research results of Zheng et al. [37]. This may be attributed to an appropriate increase in extraction temperature that could enhance molecular diffusion and promote the dissolution of TPC. However, excessive temperature would cause TPC degradation, as most polyphenols are heat-sensitive. Ming-Jun et al. proved that high temperatures were detrimental to the extraction of polyphenols [38]. Alimpia et al. [39] explored that the optimal extraction temperature for polyphenols was 60 °C, but further increases in the extraction temperature did not increase the same. Thus, 60 °C was selected as the most suitable ultrasound temperature.

### 2.3. RMS-BBD Model Fitting and Response Surface Analysis

To achieve higher extraction efficiency and retain most of the polyphenols, RSM was used to optimize the process parameters for the extraction. Multivariate regression analysis was performed using Design-Expert 13 software. The response (yield) results of the factor design are shown in Table 1, and a full quadratic regression equation is obtained as follows:

Y = 137.71 + 0.2363A + 3.76B + 12.03C + 5.38AB − 4.98AC − 3.51BC − 13.12A^2^ − 13.07B^2^ − 24.91C^2^,

where A represents the material-to-liquid ratio, B represents the ultrasonic time, and C represents the ultrasound temperature.

The response surface variance analysis is shown in Table 2. The F-value of this model was 10.01, and the *p*-value of this model was 0.0031, which indicated that this model was highly significant (*p* < 0.01). In addition, C, A^2^, B^2^, and C^2^ were all significant (*p* < 0.05). According to the equation, C had a significant effect on the TPC (*p* < 0.05), whereas A and B had no significant effect on it (*p* > 0.05). By analyzing the F-value and *p*-value, it would be determined that the factors C, A, and B have different levels of effect on the yield of *E. angustifolia* L., with C having the most significant impact, followed by B, and then A. The correlation coefficient *R*^2^ = 0.9279 suggested that the model has a good fit with the response, making it appropriate for depicting the relationship between yield and parameters. The non-significance of the lack-of-fit term (*p* = 0.2749 > 0.05) verifies the high dependability of the model.

**Figure 3:**
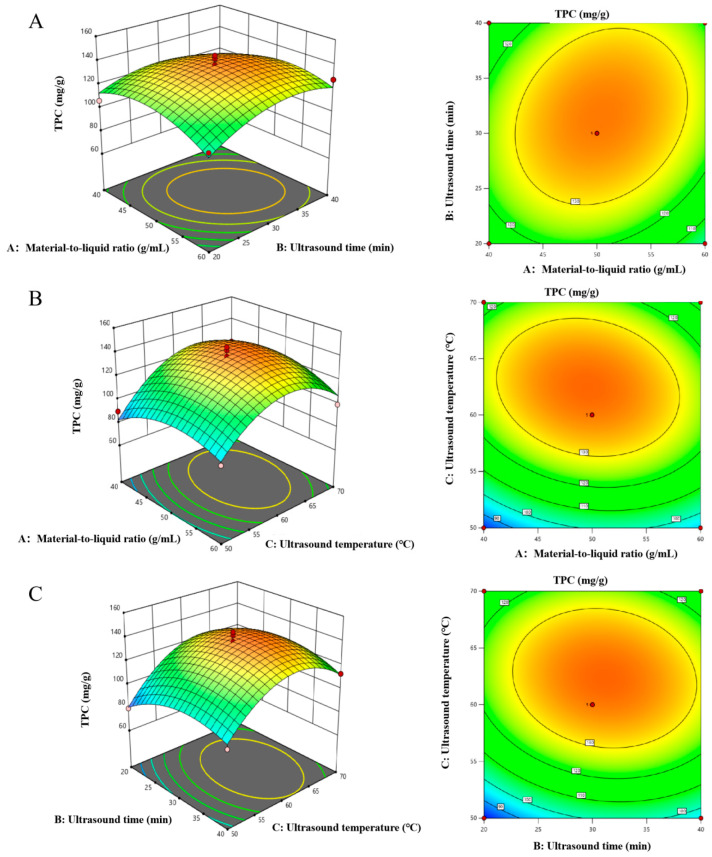
Results of the RSM. (**A**) Material-to-liquid ratio–ultrasound time. (**B**) Material-to-liquid ratio–ultrasound temperature. (**C**) Ultrasound time–ultrasound temperature, and their contour plots.

Based on the regression equations of the selected variables, response surface plots and contour plots were created (Figure 3). As shown in Figure 3A–C, with the increase in material-to-liquid ratio, ultrasound time, and ultrasound temperature, TPC first reached its peak and then decreased, which was consistent with the single-factor experiment. In addition, the interaction between material-to-liquid ratio and ultrasound time was smaller than that between material-to-liquid ratio and ultrasound temperature, as was the interaction between ultrasound time and ultrasound temperature. And that, the effect of ultrasound temperature was the biggest, and the effect of material-to-liquid ratio was the smallest. This was consistent with the analysis results in Table 2. Based on the regression model, the optimal process conditions were determined to be 49.86 g/mL, 31.10 min, and 62.35 °C. Given these conditions, the theoretical yield of *E. angustifolia* L. was calculated to be 139.33 mg/g, and the experimental yield of the *E. angustifolia* L. polyphenols was determined to be 140.30 ± 0.19 mg/g. The close alignment between the estimated and observed yields validates the accuracy of the model.

### 2.4. Comparison of Antioxidant Capacity

**Figure 4:**
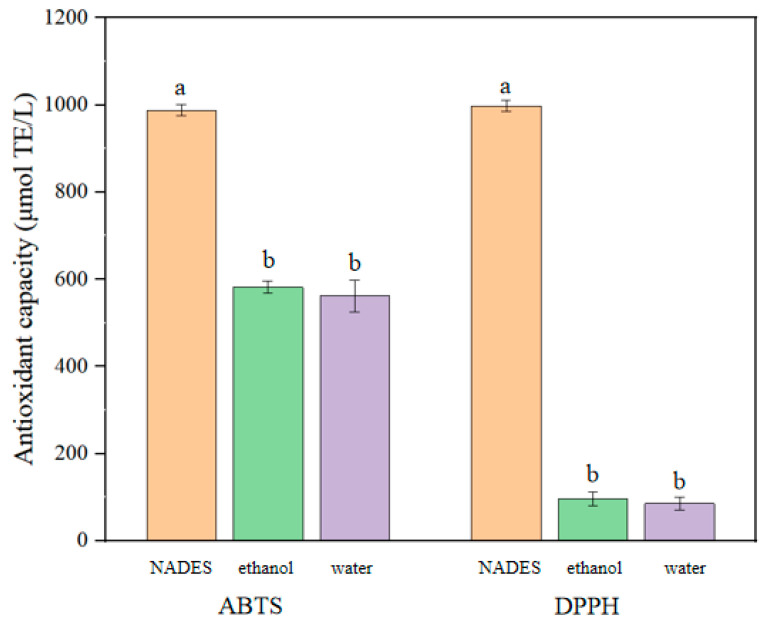
Scavenging rates of DPPH and ABTS by NADES, ethanol, and water extraction of polyphenols from *E. angustifolia* L. under the same conditions. (Different letters represent significant differences, *p* < 0.05).

The scavenging activity of polyphenol samples for ABTS and DPPH radicals is shown in Figure 4. The scavenging ability of NADES extract on two types of free radicals is significantly higher than that of the 60% ethanol group and water extraction combination extracted under the same conditions (*p* < 0.05). The ability of NADESs to clear ABTS reached 987.85 (μmol TE/g). By contrast, under the same conditions, the clearance ability of the 60% ethanol group was only 582.14 (μmol TE/g), and that of the water extraction group was only 562.15 (μmol TE/g). The ability of NADESs to clear DPPH reached 998.12 (μmol TE/g). By contrast, under the same conditions, the clearance ability of the 60% ethanol group was only 96.50 (μmol TE/g), and that of the water extraction group was only 86.12 (μmol TE/g), with a significant difference (*p* < 0.05). The results indicated that polyphenols extracted by NADES might have better free radical scavenging ability than those extracted by ethanol and water. This result may be related to the efficient extraction of polyphenols and other antioxidant components from CSP by NADES [40], which was consistent with the results of Shiling Feng (2024) [36].

### 2.5. Qualitative Analysis of E. angustifolia L. Polyphenols

The chemical composition of *E. angustifolia* L. was characterized using UPLC–IMS–QTOF–MS (Waters, Milford, MA, USA). A total of 24 compounds were detected and identified in the extract of polyphenol, including seven phenols, eight flavonoids, and four coumarins. The identification results can be found in Table 3. The primary mass spectra are attached in Figure S1. Among them, components such as oxyphyllacinol and citroenol [41,42] were reported to have certain antioxidant and anti-inflammatory effects. Hnit et al. [43] found that agrimol B, a polyphenol extracted from Agrimonia pilosa Ledeb, has anticancer properties and physiological activity. Fási et al. [44] pointed out that methyl caffeate could induce the production of effective anti-tumor metabolites. It would be seen that the polyphenols from *E. angustifolia* L. contain many functional and active ingredients. This was conducive to expanding the application of *E. angustifolia* L. in the fields of medicine and food.

## 3. Materials and Methods

### 3.1. Material and Chemicals

*E. angustifolia* L. samples were collected in Xinjiang in 2023. *E. angustifolia* L. is recorded by the Beijing Natural History Museum as IBSC170466. The collected samples were dried in a convection oven at 50 °C for 24 h to a constant weight. Subsequently, samples that were pulverized with the help of a grinder were used through a 60-mesh screen.

Choline chloride (99%), phenol, 1,1-diphenyl-2-picryl-hydrazil (DPPH), and 2,2′-azinobis-(3-ethylbenzthiazoline-6-sulphonate) (ABTS) were obtained from Macklin. In addition, gallic acid, quercetin, and Trolox standards were provided by Sigma. Anhydrous ethanol, urea, ethylene glycol, malic acid, glycerol, ammonium acetate, malonic acid, 1,2-propanediol, 1,4-butanediol, and dl-lactic acid were provided by China National Pharmaceutical Group Chemical Reagent Co., Ltd. (Shanghai, China).

### 3.2. Preparation of NADES

To screen out the optimal NADES with high efficiency, of the nine kinds of NADESs mentioned above, 60% ethanol and water were used in this process. The detailed process was as follows: NADESs could be prepared by weighing an appropriate amount of the reactive substance according to Table 4 and placing it in a 100-mL beaker, stirring it with a magnetic stirrer at 80 °C until a colorless transparent liquid was obtained, and then cooling to a room temperature of 25 °C. To reduce the viscosity of the resulting liquid, 20% (volume fraction) of water was added. The hydrogen bond donors and acceptors were mixed in the molar ratios recommended by the relevant references [45,46] shown in Table 4. The solvent was stored in glass bottles at room temperature.

### 3.3. Extraction Procedure

*E. angustifolia* L. powder was blended with a variety of NADES (Table 4), 60% ethanol, and water. The mixture was then subjected to ultrasound extraction (Ultrasonic Cleaner, JP-060S, Shenzhen Jiemeng Cleaning Equipment Co., Ltd., Shenzhen, China). Perform ultrasonic treatment at a set temperature and time (50 °C, 30 min). Afterward, the sample was subjected to centrifugation at 8000 rpm for 5 min (high-speed centrifuge, TGL-20bR, Shanghai Anting Scientific Instrument Co., Ltd., Shanghai, China).

### 3.4. Determination of Total Phenolic Content in the Extracts

The determination of total phenolic content (TPC) was performed using Folin-Ciocalteu colorimetric method as described in a previous study [47]. A mixture of 125 mL of Folin–Ciocalteu reagent and 50 μL of sample extracts was combined, followed by incubation at room temperature for 6 min. Subsequently, we add 1.25 mL of a 7% Na_2_CO_3_ solution and make up to 3 mL with double-pure water, followed by incubation in a 40 °C water bath for 90 min. The optical density of each sample was determined by a UV–vis spectrophotometer at a wavelength of 760 nm (UV spectrophotometer, J6, Shanghai Jinghai Technology Instrument Co., Ltd., Shanghai, China). TPC was assessed and expressed as milligrams of gallic acid equivalents per gram (mg GAE/g). A calibration curve was employed to derive a mathematical equation that precisely depicted this correlation: Y = 0.0021X + 0.0079 (*R*^2^ = 0.9965).

### 3.5. Experimental Design

#### 3.5.1. Single Variable Experiment

With NADES-3 as the extraction solvent, a single-variable experiment was performed to evaluate the effects of various extraction times (10, 20, 30, 40, 50, and 60 min), temperatures (20, 30, 40, 50, and 60 °C), and material-to-liquid ratios (1:30, 1:40, 1:50, 1:60, and 1:70 g/mL) on the extraction rate of total phenol in *E. angustifolia* L.

#### 3.5.2. RSM Using BBD

The material-to-liquid ratio (A), ultrasound time (B), and ultrasound temperature (C) were optimized using the response surface methodology. TPC yield from *E. angustifolia* L. extracts served as the response variable. The three factors and their respective three levels, as outlined in Table 5, were considered for optimization to establish the ideal extraction conditions, which were then applied in subsequent experiments.

### 3.6. Antioxidant Activity

#### 3.6.1. DPPH Radical Scavenging Activity

DPPH radical scavenging activity was determined as described by Shopska et al. [48]. Briefly, 100 μL of sample was added to 3.9 mL of 12.5% DPPH solution. The mixture was allowed to react for 20 min in the dark, and then the absorbance at 517 nm was determined. A standard solution of 0–1000 μmol/L Trolox was prepared, and a standard curve was prepared for calculating the DPPH radical scavenging activity assay. The standard curve regression equation was obtained: Y = −0.0006X + 0.6459, *R*^2^ = 0.9946. The results were expressed as µmol Trolox/L.

#### 3.6.2. ABTS Radical Scavenging Activity Assay

The ABTS assay was based on the method of Anna Floegel (2011) [49], with slight modifications. Briefly, 5 mL and 88 µL of 7 × 10^−3^ mol/L ABTS solution and 0.14 mol/L potassium persulfate solution, respectively, were added, and the mixture was placed in the dark for 12–16 h. The mixture was diluted with methanol until the absorbance was 0.70 ± 0.02 at 734 nm. Afterward, 0.1 mL of sample was added to 3.9 mL of ABTS solution for 8 min in the dark, and the absorbance value was measured at 734 nm. A standard solution of 0–1000 μmol/L Trolox was prepared, and a standard curve was prepared for calculating the ABTS radical scavenging activity assay. The standard curve regression equation was obtained: Y = −0.0007X + 0.7015, R^2^ = 0.9981. The results were expressed as µmol Trolox/L.

### 3.7. Identification of Phenolic Compounds Using UPLC–IMS–QTOF–MS Analysis

The polyphenol of *E. angustifolia* L. was determined, followed by Li et al. (2019) [50] with slight modifications. In brief, the ultraperformance liquid chromatography (UPLC) I-Class system was coupled with ion mobility spectrometry (IMS) and quadrupole time-of-flight mass spectrometry (QTOF–MS) (Waters, Milford, MA, USA). An ACQUITY UPLC-BEH C18 column, 2.1 mm inner diameter × 100 mm and 1.7 μm particle size, was used. HPLC grade with 0.1% formic acid (*v*/*v*) and acetonitrile (*v*/*v*) were used as solvents A and B, respectively. The injection volume was 5 μL, the flow rate was 0.3 mL/min, and the column temperature was 25 °C. The elution was conducted at 95% of solvent A and 5% of solvent B at the beginning, increased via linear gradient to 90% B at 16 min, 75% at 25 min, and 100% at 17 min. The post- and pre-injection wash took 5 min.

The high-definition MS^E^ was conducted with the optimized parameters: capillary voltage at 2.5 kV, source temperature at 120 °C, and desolvation temperature at 500 °C. The gas flow rate in the conical hole is 50 L/h. The mass range was from *m*/*z* 50–2000 with a 0.2 s/scan. Data were processed using Waters Progenesis QI database scientific information system software (UNIFI software).

### 3.8. Statistical Analysis

All results should be repeated at least three times. Design-Expert 13 software was utilized to create the experimental design for the response surface analysis aimed at refining the extraction methodology. IBM SPSS Statistics 20 software was used for statistical analysis. Differences were considered significant at the level of *p* < 0.05.

## 4. Conclusions

In the present study, the ultrasound-assisted NADES technique was effectively used to obtain polyphenols from *E. angustifolia* L. Furthermore, malonic acid was determined to be the most suitable solvent for the extraction of polyphenols from *E. angustifolia* L. Based on the RSM results, the optimal conditions are the following: 49.86 g/mL of material-to-liquid ratio, 31.10 min of ultrasound time, and 62.35 °C of ultrasound temperature, resulting in a high yield of 140.30 ± 0.19 mg/g. The antioxidant experiment showed that the removal efficiency of DPPH and ABTS by polyphenols from *E. angustifolia* L. extracted with NADS solvent was significantly higher than that extracted with traditional solvents such as ethanol and water. Finally, its possible 24 polyphenolic compounds were determined by UPLC–IMS–QTOF–MS. This study contributes to the development and utilization of polyphenols from *E. angustifolia* L.

## Figures and Tables

**Table 1:** Experimental design for ultrasound-assisted extraction.

No.	A: Material-to-Liquid Ratio, g/mL	B: Ultrasound Time, min	C: Ultrasound Temperature, °C	Yield (mg/mL)
1	−1	−1	0	106.32
2	0	0	0	145.76
3	−1	0	−1	89.92
4	0	−1	1	115.29
5	1	0	1	99.49
6	0	0	0	129.57
7	0	0	0	138.14
8	1	0	−1	90.03
9	0	1	1	112.9
10	−1	0	1	119.28
11	0	−1	−1	79.55
12	0	1	−1	91.21
13	1	1	0	127.49
14	0	0	0	143.14
15	−1	1	0	105.94
16	0	0	0	131.95
17	1	−1	0	106.34

**Table 2:** Analysis of variance for regression model equation.

Source	Sum of Squares	Degree of Freedom	Mean Square	F-Value	*p*-Value
Model	6003.37	9	667.04	10.01	0.0031
A	0.4465	1	0.4465	0.0067	0.9370
B	112.80	1	112.80	1.69	0.2344
C	1158.01	1	1158.01	17.38	0.0042
AB	115.89	1	115.89	1.74	0.2287
AC	99.00	1	99.00	1.49	0.2623
BC	49.35	1	49.35	0.7406	0.4180
A²	725.16	1	725.16	10.88	0.0131
B²	718.82	1	718.82	10.79	0.0134
C²	2612.35	1	2612.35	39.21	0.0004
Residual	466.43	7	66.63		
Lack of Fit	272.52	3	90.84	1.87	0.2749
Pure Error	193.91	4	48.48		
Cor Total	6469.79	16			
R^2^	0.9279				

**Table 3:** Chemical components of polyphenol extract of *Elaeagnus angustifolia* L.

ID	RT (min)	Observed [M−H]− *m*/*z*	Response	Chemical Formula	Component Name	Type
1	0.81	379.0834	192	C_20_H_14_O_5_	Sophoracoumestan A	Coumarin
2	8.63	853.461	129	-	Pomodic acid 3-β-O-α-L-2′-Acetoxypyranoarabinyl-28-O-β-D-glucopyranose ester	Flavonoids
3	9.21	935.5035	239	C_14_H_24_O_8_	Marsdekoiside B,2	Flavonoids
4	10.93	421.1868	178	C_21_H_28_O_6_	Octahydrocurcumin	Metabolites of curcumin
5	11.38	401.0871	13935	C_19_H_16_O_7_	6-Aldehydoisoophiopogonanone A	Flavonoids
6	11.78	207.1029	207	C_12_H_16_O_3_	β-Asarone	Phenols
7	12.56	239.1289	283	C_12_H_10_O_3_	Cnidiumlac	Coumarin
8	12.9	587.3597	601	C_34_H_52_O_8_	Quinatoside A	Flavonoid glycoside
9	15.99	347.1713	12607	-	Schizonepetoside E	Phenolic glycoside
10	16.92	681.2966	21984	C_37_H_46_O_12_	Agrimol B	Phenols
11	17.04	297.1529	2846	C_19_H_22_O_3_	Ostruthins	Coumarin
12	17.05	359.1534	11376	C_15_H_16_O_4_	Citroenol	Coumarin
13	17.13	239.0591	3553	C_10_H_10_O_4_	Methyl caffeate	Phenols
14	17.18	464.0986	179	C_21_H_21_O_11_	Delphinidin-3-glucoside	Flavonoids
15	17.86	387.0961	3726	-	Caffeic acid-β-D-glucopyranoside	Phenolic acid
16	17.91	391.2084	179	C_22_H_32_O_6_	Picrasinol B	Phenols
17	17.94	359.1829	949	C_20_H_26_O_3_	Oxyphyllacinol	Flavonoids
18	18	671.1424	128	-	Quercetin 7-O-[β-D-glucopyranose group (1 → 6)-β-D-glucopyranoside	Flavonoids
19	18.06	337.236	1115	C_20_H_34_O_4_	Kirenol	Phenols
20	18.13	553.2432	709	C_30_H_36_O_7_	Kushenol M	Flavonoids
21	18.16	533.1563	159	C_17_H_14_O_3_	Draconin	Anthraquinone
22	18.26	223.0278	146563	C_9_H_6_O_4_	5,7-dihydroxychromogen ketone	Phenols
23	18.44	297.0725	243	C_17_H_14_O_5_	5-hydroxy-7,4′-dimethoxyflavonoid	Flavonoids
24	18.66	675.2257	322	C_25_H_28_O_4_	AMulberrofuran A	Phenols

**Table 4:** NADESs prepared.

No.	Solvent Abbreviation	HBA	HBD	Molar Ratio	Moisture Content
1	NADES-1	Choline chloride	Malic acid	1:1	20%
2	NADES-2		Propylene Glycol	1:2	
3	NADES-3		Malonic acid	1:2	
4	NADES-4		Ethylene glycol	1:2	
5	NADES-5		Ammonium acetate	1:2	
6	NADES-6		Glycerol	1:2	
7	NADES-7		Butanediol	1:2	
8	NADES-8		Urea	1:2	
9	NADES-9		Lactic acid	1:2	

**Table 5:** Variables coding levels and actual values of RSM-BBD.

Variables	Levels		
	−1	0	1
A(material-to-liquid ratio, g/mL)	1:40	1:50	1:60
B(ultrasound time, min)	20	30	40
C(ultrasound temperature, °C)	50	60	70

## Data Availability

Data are contained within the article and Supplementary Materials.

## Supplementary Materials

The following supporting information can be downloaded at: https://www.mdpi.com/article/10.3390/molecules29112412/s1, Figure S1: Mass spectrum of polyphenol extract from *Elaeagnus angustifolia* L.
