# Supplementary figures and images for: Green Extraction of Polyphenols from Elaeagnus angustifolia L. Using Natural Deep Eutectic Solvents and Evaluation of Bioactivity
Source: Molecules. 2024 May 21;29(11):2412. doi: 10.3390/molecules29112412 (PMC11173772; doi:10.3390/molecules29112412)

## Supplementary materials

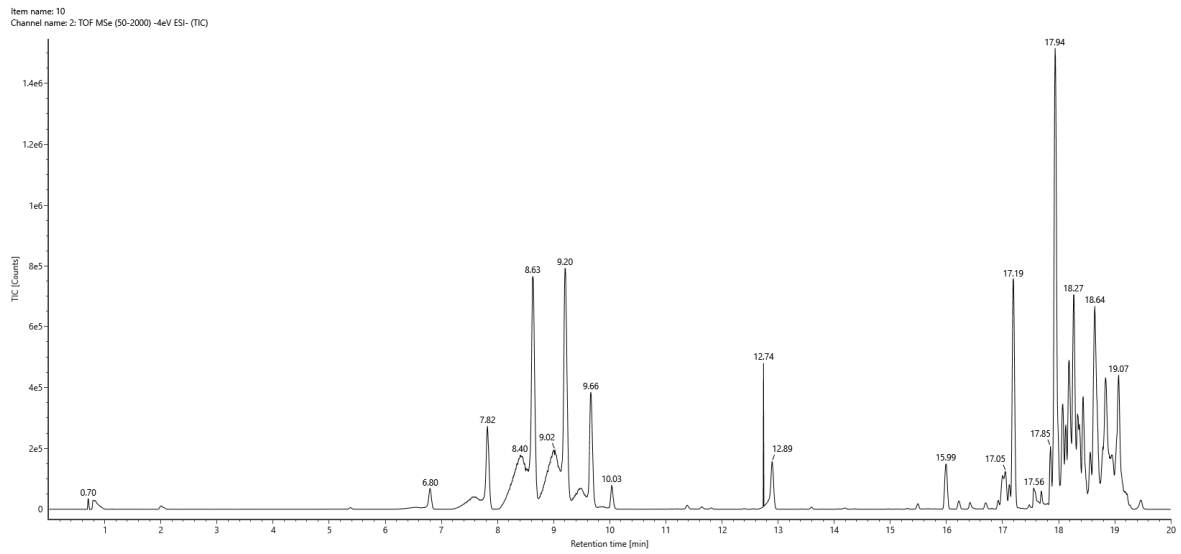

**Figure S1.** Mass spectrum of polyphenol extract from *E. angustifolia* L.

Supplement: Supplementary file 1 [file molecules-29-02412-s001.zip › molecules-2999249-supplementary.pdf]
